# Hepatocytes as Model for Investigating Natural Senotherapeutic Compounds and Their Effects on Cell Cycle Dynamics and Genome Stability

**DOI:** 10.3390/ijms26146794

**Published:** 2025-07-16

**Authors:** Anastasia Fizikova, Anna Prokhorova, Daria Churikova, Zahar Konstantinov, Roman Ivanov, Alexander Karabelsky, Stanislav Rybtsov

**Affiliations:** 1 Research Center for Translational Medicine, Sirius University of Science and Technology, Olympic Ave. 1, 354340 Sochi, Russia; liprohorova@yandex.ru (A.P.); daria.tchurikova@yandex.ru (D.C.);; 2 Department of Genetics and Biotechnology, Saint Petersburg State University, 7/9 Universitetskaya Emb., 199034 Saint Petersburg, Russia; zakhar.konstantinov@mail.ru

**Keywords:** cell cycle, senolytics, senomorphics, SASP, CRISPR/Cas, prime-editing, hepatocyte, hemophilia B, cancer

## Abstract

DNA is inherently unstable and is susceptible to damage from both endogenous sources (such as reactive oxygen species) and exogenous factors (including UV, ionizing radiation, and chemicals). The accumulation of DNA damage manifests as genetic mutations, chromosomal instability, and the stalling of DNA replication and transcription processes. Accumulated DNA damage influences apoptosis and cell cycle checkpoints, serving as one of the key triggers for the manifestation of the senescent phenotype. Both aging and cancer are associated with the accumulation of mutations in somatic cells. Disruption of cell cycle control and uncontrolled proliferation are fundamental characteristics of any cancer cell, with the majority of anticancer drugs acting as inhibitors of cyclin-dependent kinases, thereby inducing a transition of cells into a senescent state. Consequently, disturbances in the dynamics and regulation of inflammatory responses, oxidative stress, cell proliferation, DNA damage repair, and epigenetic anomalies, along with the influence of retroviruses and transposons, lead to the accumulation of senescent cells within the human body, characterized by blocked replication and cell cycle, as well as a distinct secretory phenotype. The age-related or disease-associated accumulation of these senescent cells significantly alters the physiology of tissues and the organism as a whole. Many secondary metabolites of higher plants exhibit senolytic and senomorphic activities, although most of them are not fully characterized. In this review, we will explore the principal signaling pathways in mammalian cells that govern the cell cycle and cellular senescence, with a particular emphasis on how their dynamics, expression, and regulation have been modified through the application of senotherapeutic compounds. The second section of the review will identify key target genes for the metabolic engineering, primarily aimed at enhancing the accumulation of plant secondary metabolites with potential therapeutic benefits. Lastly, we will discuss the rationale for utilizing liver cells as a model system to investigate the effects of senolytic compounds on human physiology and health, as well as how senotherapeutic substances can be leveraged to improve gene therapy approaches based on CRISPR/Cas9 and prime-editing technologies.

## 1. Introduction

DNA damage affects most aspects of the aging phenotype and is considered the most likely cause of aging [1]

**Figure 1:**
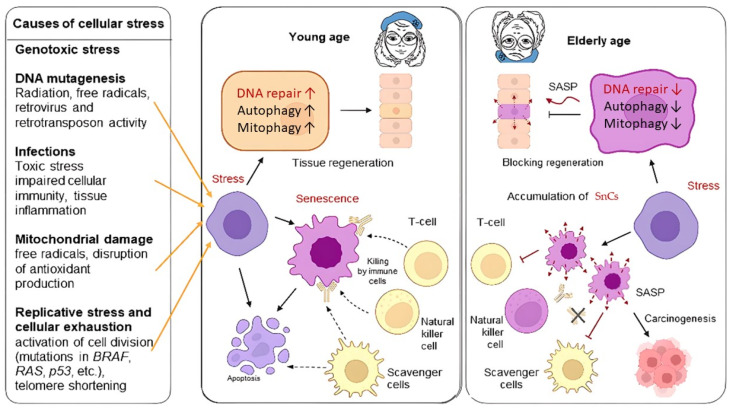
Age-related changes in the response of mammalian cells to stress and the role of cellular senescence.

In cells that, due to external or internal damage, are unable to undergo programmed cell death (apoptosis), a protective molecular mechanism of accelerated aging (senescence) is initiated. Such cells are termed senescent (SnCs): they cease to divide, autophagy (the ability of cells and tissues to self-cleanse) is suppressed, the capacity for the clearance of damaged mitochondria (mitophagy) is diminished, antioxidant production is impaired, and they produce factors that not only damage the cells themselves but also the surrounding tissues. Additionally, carbohydrate metabolism is activated, sustaining a pro-inflammatory secretory phenotype known as the Senescence-Associated Secretory Phenotype (SASP). Senescent cells, through the secretion of SASP, induce local inflammation and attract the immune system to eliminate aged, non-functional cells. With age, this process becomes less effective primarily due to the aging of the immune system [2,3] (Figure 1).

The accumulation of senescent cells leads to “sterile” chronic inflammation, impaired regeneration, and dysfunction of internal organs, inflammatory obesity, and an increased risk of oncogenesis [4,5,6,7]. In a young organism, cells can manage stress through DNA repair, autophagy, mitophagy, and intracellular immunity; however, when they are unable to rectify the consequences of stress, apoptosis is triggered. The blockade of apoptosis (e.g., during viral infections) may lead to cellular senescence. In youth, immune cells destroy or label SnCs for phagocytosis during inflammation. Accumulation of SnCs with an inflammatory phenotype, diminished tissue regeneration, and reduced production of naïve immune cells. The number of exhausted immune and senescent cells increases, leading to decreased antibody secretion, which impairs tissue clearance of SnCs and exacerbates inflammation [7,8]. In older age, SnCs are more likely to become senescent due to reduced autophagy, mitophagy, and repair mechanisms.

One of the triggers leading to the transition of cells to a senescent state is the accumulation of DNA damages [9]. DNA damage prevents accurate replication, controlled transcription, and safe storage of genetic information [1].

DNA integrity can only be maintained through continuous, effective repair. Most long-lived species are characterized by duplication or high expression level of repair genes [10,11,12]. Inherited defects in systems that maintain genomic integrity not only predispose to cancer but also underlie numerous segmental forms of premature aging, suggesting a close link between genomic integrity, cancer, and aging [1,13].

## 2. Nuclear and Mitochondrial Genome Damage: Implications for Aging and Disease

Nuclear and mitochondrial genomes are constantly subjected to damage from both exogenous and endogenous inducers. Examples of exogenous sources include ultraviolet (UV) radiation, X-rays, and chemical compounds, while endogenous sources encompass reactive oxygen species (ROS), glycation products, aldehydes, and spontaneous hydrolysis reactions. The molecular consequences of accumulating DNA damage over time include mutations, chromosomal instability, and stalling of RNA and DNA polymerases due to DNA lesions. Mutations typically have detrimental effects on tissue and organ functions and are a primary cause of cancer and genetic disorders. DNA damage, rearrangements, chemical modifications of nucleotides, and the accumulation of abnormal secondary structures and intermediates hinder accurate replication, regulated transcription, and reliable storage of genetic information. Thus, the importance of effective DNA repair systems cannot be overstated. Inherited defects in DNA repair and maintenance systems not only predispose individuals to cancer but also underlie various forms of premature aging, highlighting the close relationship between genome integrity, cancer, and aging [13].

### 2.1. Progeroid Syndromes and DNA Repair Defects

Werner syndrome manifests early with osteoporosis, cataracts, type 2 diabetes, and graying of hair in individuals under 30 years of age. Werner, along with Bloom and Rothmund–Thomson syndromes, are associated with mutations in RecQ helicase family genes, which normally participate in the unwinding of recombination intermediates, replication, and telomere repair. Another condition, Cockayne syndrome, is characterized by early-onset atherosclerosis, osteoporosis, neurodegeneration, and photosensitivity, resulting from mutations in the *ERCC6* and *ERCC8* genes, whose products are involved in nucleotide excision repair (NER). Nijmegen syndrome and Hutchinson–Gilford progeria are attributed to defects in the repair of double-strand breaks (DSBs) in DNA, explained by dysfunctions in the proteins Nibrin and Lamin [13,14,15,16].

### 2.2. Telomere Shortening

Critically short telomeres activate the DNA damage response (DDR), resulting in cellular senescence. DNA repair processes induce chromatin remodeling, while chromatin structure influences susceptibility to DNA damage and accessibility for repair systems. In mammals, telomeres consist of thousands of TTAGGG repeats, covered by the shelterin complex, which promotes the formation of a T-loop structure, thereby concealing the telomeric end and preventing the activation of DDR sensors [17]. Due to incomplete synthesis of the lagging strand during DNA replication, the number of repeats decreases with each cell division. In embryos and some somatic stem cells, this loss is compensated for by telomerase, which is suppressed in most somatic cells during early development, limiting the number of cell divisions until telomeres become critically short. Uncontrolled telomerase activity leads to chronic DDR activation, resulting in replicative senescence. The occurrence of even a single DSB is sufficient to cause complete cell cycle arrest [18]. Telomere shortening during aging serves as an alternative to uncontrolled proliferation and, consequently, tumor formation [19]. The assessed length of telomeres in human tissues does not indicate that, on average, telomeres become critically short during normal aging, even in elderly individuals [20]. However, it is presumed that telomere shortening may alter the expression of subtelomeric genes, which may contribute to the development of aging phenotypes [21].

### 2.3. Epigenetic Changes

DNA damage is a primary factor in age-related epigenetic changes. DNA methyltransferase Dnmt1 localizes to DNA repair sites, and chromatin undergoes remodeling and regulates repair, leaving epigenetic marks [22]. In human cells, DDR leads to the loss of trimethylation of lysine 27 on histone 3 (H3K27me3) and the formation of phosphorylated histone γH2AX at DSBs, which accumulate with age, indicating chromatin changes. Single-cell sequencing of human neurons has confirmed that DNA SCARS found in senescent cells represent permanent chromatin changes due to irreparable damage [23]. Poly(ADP-ribosyl)ation of histones and the PARP1 protein facilitate the repair of single-strand breaks but reduce NAD+ levels, which can trigger apoptosis and affect Sirtuin proteins, altering chromatin acetylation and gene expression [24]. Continuous DNA damage leaves epigenetic marks, contributing to intercellular heterogeneity in aging, making transcription in old cells variable and unstable [23].

### 2.4. Protein Misfolding

Several aging-related diseases are also associated with protein misfolding and aggregation due to increased transcriptional stalling and transcriptional noise as a consequence of epigenetic changes in the genome, such as Alzheimer’s and Parkinson’s diseases and Cockayne syndrome [25]. Single-cell sequencing has shown that somatic mutations increase with age and occur at a higher rate in patients with neurodegenerative diseases [25]. In progeroid mice with mutations in NER genes, accelerated declines in gene transcription provoke proteotoxic stress and protein aggregates due to the loss of stoichiometry in protein complexes [26,27,28]. Defects in chaperones, the ubiquitin-proteasome system, and autophagy can also lead to the accumulation of misfolded proteins. In mouse models with DNA repair deficiencies and active DDR cascades, the induction of key regulators of the endoplasmic reticulum stress response and autophagy, such as IRE1α and the transcription factor XBP1, is essential for survival under conditions of persistent DNA damage [27]. Unrepaired DNA damage leads to the development of a chronic senescence-associated secretory phenotype and overloads the unfolded protein response in the endoplasmic reticulum (UPRER), while caloric restriction alleviates transcriptional stress and facilitates UPRER function [29]. Therefore, the primary causes of proteotoxic stress associated with aging are considered to be DNA damage and epigenetic changes in chromatin.

### 2.5. Mitochondria Function Defects

Senescent cells, which accumulate with age, are characterized by impaired mitochondrial function, resulting in reduced ATP levels and increased concentrations of reactive oxygen species, as well as disrupted regulation of mitophagy. Such conditions are also characteristic of many diseases, including Alzheimer’s and Parkinson’s diseases, cardiovascular diseases, diabetes, and cancer [15]. Previously, age-related mitochondrial dysfunction was attributed to the accumulation of somatic mutations in mtDNA due to replication errors and the absence of effective repair systems in organelles. Supporting evidence came from studies showing that mice with defective DNA polymerase (POLG) exhibited increased mutations and signs of premature aging, as well as a correlation between the loss of cytochrome C oxidase (COX) activity in aging tissues and increased frequencies of mtDNA mutations [30,31]. However, modern methods such as digital PCR and deep sequencing have demonstrated a lack of age dependence in the frequencies of mitochondrial DNA mutations. Therefore, it is now more likely that mitochondrial dysfunction may arise from damage and alterations in nuclear DNA function. For instance, it is known that aging activates PARP1, depletes NAD+, and disrupts mitophagy, while PARP inhibition or NAD+ supplementation may improve mitochondrial functions and mitigate phenotypes of premature aging [32]. Notably, interesting results have been obtained from model eukaryotic organism, such as *Saccharomyces cerevisiae* yeast, where the functioning of organelles and the stability of the mitochondrial genome can be distinctly traced in relation to mutations in nuclear-localized genes. Since yeast are facultative anaerobes and maintain viability upon complete loss of the mitochondrial genome, this model is convenient for studying the mechanisms of mutual regulation of mitochondrial and nuclear genome stability depending on the metabolic state of the cells and the stage of the cell cycle. For example, yeast studies have demonstrated disrupted distribution of mitochondrial nucleoids on the background of nuclear *PHO85* gene mutations, as well as the dependence of mitochondrial genome stability on the metabolic state of the cells [33,34,35]. The cyclin-dependent protein kinase Pho85 is a homolog of human CDK5; in *Drosophila*, it has been shown that increased Cdk5 activity is associated with accelerated aging, while reduced kinase activity slows this process [36]. The interplay of metabolism, cell cycle regulation, aging, and the development of the senescent phenotype illustrates the physiological mutation theory proposed by Lobashev over 80 years ago [37].

### 2.6. Calorie Restriction

It is now known that dietary changes impact aging and lifespan across the entire animal kingdom: caloric restriction is the most striking example of how metabolic changes through the suppression of Insulin/Insulin-like Growth Factor Signaling (IIS), activation of sirtuins, and inhibition of mTOR lead to increased NAD+, reduced ROS, and decreased levels of DNA damage [38]. In progeroid mice and worms, a weakening of IIS occurs due to the phosphorylation of key proteins in the IIS-mTOR pathways by the DDR kinase ATM [39]. In vivo studies have shown that mTOR suppression by rapamycin increases levels of the DNA repair protein O-6-methylguanine-DNA methyltransferase (MGMT) and activates Sirt1 and AMPK, facilitating DNA damage repair through enhanced NER efficiency and epigenetic adaptation [40]. The protein kinase AKT is considered as a central positive regulator of various pathways that perceive nutrient composition and quantity: it negatively regulates DNA repair and inhibits key DDR factors, including Chk1, Topbp1, and p53 [41]. The transcription factor FOXO3a, activated by reduced IIS, promotes the binding of TIP60 to ATM, optimizing ATM activation following DNA damage. FOXO3a regulates cellular fate under stress conditions, inducing cell cycle arrest and apoptosis, influencing glucose metabolism, and playing a role in protection against oxidative stress, including tumor suppression and longevity [42].

## 3. Senescent Phenotype of Cells

**Figure 2:**
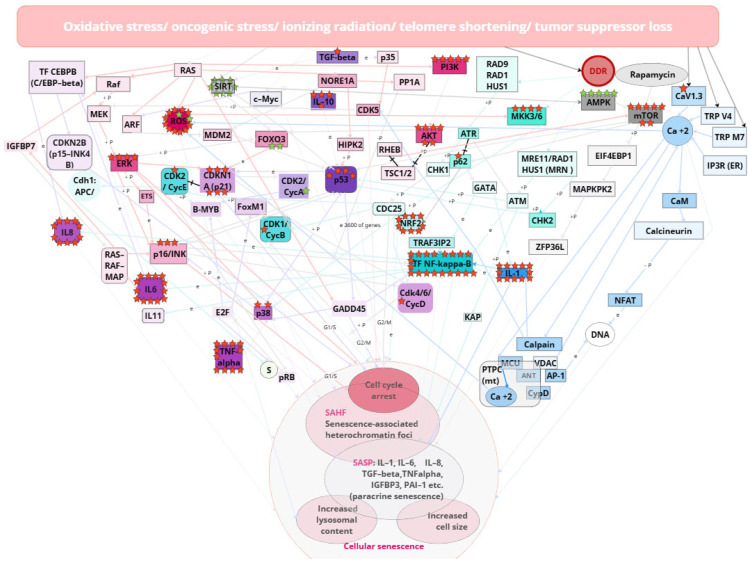
Simplified diagram of the mammalian signaling network involved in the age-associated secretory phenotype (SASP) (according to the KEGG database: https://www.kegg.jp/entry/map01110 (accessed on 4 April 2025)): The calcium-signaling pathway is indicated in blue, the mTOR/DDP signaling pathway in gray, the NF-kB signaling pathway in turquoise, and the Ras-ERK and MAPK/ERK signaling pathways in pink. The ‘e’ represents expression regulation, ‘+P’ indicates phosphorylation, and ‘−P’ denotes dephosphorylation. The known effects of different natural compounds with senotherapeutic activity are marked with stars: red indicates suppression, while green signifies activation.

Cellular senescence can be triggered by increased concentrations of free radicals, heightened expression of oncogenes, genotoxic stress, ionizing radiation, DNA damage, telomere shortening/damage, mitochondrial dysfunction, chromatin structure disruption, epigenetic factors, as well as the presence of viral nucleic acids and the activity of retroviruses and transposons [43] (Figure 2).

### 3.1. Calcium Signaling Pathway

Cellular senescence, a process where cells permanently stop dividing, intracellular calcium levels generally increase. This elevation can be attributed to calcium influx through the plasma membrane or release from intracellular stores like the endoplasmic reticulum and mitochondria. The increased calcium levels appear to play a role in the establishment and maintenance of senescence, potentially by influencing downstream signaling pathways. Calcium signaling is linked to several key pathways involved in senescence, such as the DNA damage response (DDR), the p53/p21/RB pathway, and is involved in the development of the senescent phenotype [43]. Manipulating calcium levels, for example, by using calcium chelators like BAPTA, can affect the progression of senescence, suggesting that the calcium increase is not just a consequence but also a driver of the senescent state. While increased calcium can promote senescence, it can also trigger apoptosis in normal cells when levels are too high. The outcome likely depends on the specific cell type, the magnitude and duration of the calcium elevation, and the presence or absence of key senescence-related proteins like p53 and RB [44].

Transient SnCs secrete SASP, initiating remodeling processes and tissue regeneration, and are removed by immune cells. The activation of the senescence program depends on the expression of cytoplasmic DNA damage sensors such as cGMP-AMP (cGAMP) synthase (cGAS) [43].

### 3.2. DDR, ATR, ATM, p53, and NF-kB-Mediated Pathway

For instance, following DNA damage or proliferative stress, the activation of DNA Damage Response (DDR) pathways occurs, marked by increased expression of γH2AX, a marker of this signaling pathway. The increase in SnCs numbers is also associated with reduced expression of endogenous reverse transcriptase inhibitors, which elevates the systemic level of retrotransposon and retroviral activity, increasing the risk of DNA damage and subsequently triggering the DDR signaling pathway and activation of the senescence program [45,46]. The appearance of a high quantity of phosphorylated p53 leads to the activation of the p21 factor, promoting cell cycle arrest in the G0 phase; cells become “positive” for p16ink4 markers and “negative” for proliferation markers Ki67 and phospho-Rb. Additionally, due to DNA damage, 53BP1 is recruited to sites of DNA damage in the nucleus (which plays a crucial role in DNA repair). Characteristic changes in cell morphology are observed, leading to chronic inflammation, obesity, internal organ dysfunction, and frequently oncogenesis at the organismal level [47].

One of the primary intracellular pathways responsible for inducing inflammatory responses and SASP is the NF-kB-mediated pathway [48,49]. NF-κB acts as a master regulator of the SASP: p16 directly binds to p65 and inhibits the nuclear factor kappa light chain enhancer of activated NF-κB complex. This interaction serves as a brake on NF-κB activity, which helps prevent its pro-oncogenic functions. When p16 is active, it binds to p65 and prevents it from interacting with other components required for NF-κB activation. As a result of this inhibition, NF-κB activity and its ability to trigger transcription of inflammation- and SASP-related genes are reduced. Thus, p16 may influence NF-κB, reducing its activity and therefore helping to maintain cellular stability and prevent cells from transforming into tumor cells. When p16 is lost, for example, in tumor cells, NF-κB can become overactive, leading to increased secretion of pro-inflammatory factors and promoting oncogenesis. Thus, p16 plays an important role in the regulation of NF-κB and, consequently, in the control of cell senescence and tumor transformation processes [50,51].

In the induction of senescence, NF-κB often acts in concert with p53: p53, known as the ‘guardian of the genome’, is activated in response to DNA damage and stresses, including oxidative stress. It promotes cell-cycle arrest, apoptosis and SASP activation. NF-κB, on the other hand, is activated in response to inflammatory signals and plays an important role in the regulation of immune response and inflammation. It can also promote senescence but in some contexts can support cell proliferation and survival. p53 can inhibit NF-κB activity by interfering with its pro-oncogenic functions. For example, p53 can inhibit transcription of genes regulated by NF-κB, resulting in reduced inflammation. At the same time, NF-κB can enhance p53 expression in response to certain stimuli, which can lead to activation of cell arrest and senescence programs. Depending on cell type and conditions, one of these proteins may dominate the regulation of senescence. For example, in some tumor cell types, p53 may be inactivated, allowing NF-κB to exert its pro-oncogenic functions. In other cells, especially normal somatic cells, p53 may be more active and contribute to more pronounced senescence, whereas NF-κB may play a supporting role [49].

### 3.3. SASP

Currently, around fifteen hundred proteins associated with SASP have been described (Figure 2). The accumulation of aging cells in tissues above a threshold value significantly alters normal functioning. These proteins are involved in the pathogenesis of cardiovascular, neurodegenerative diseases, and diabetes [8,46]. The role of senescent cells as a niche for tumorigenesis is also being discussed [52]. The spectrum of SASP proteins varies across different types of malignant tumors, depending on the type of primary inflammatory inducer. The accumulation of senescent cells is associated with diseases such as atherosclerosis and cardiovascular diseases [53], neurodegenerative disorders including Alzheimer’s disease [54], osteoporosis and osteoarthritis [55,56], steatosis [57], trisomy 21 [9], adipose tissue atrophy, muscle degeneration, and cataracts, where selective elimination of senescent cells has already shown promising results [57].

Notably, universal SASP proteins, whose increased secretion is observed in many tumor types, account for 9–23% [58]. A correlation has been demonstrated between severe cases of viral infections, such as COVID-19, and high senescence levels [45]. The primary molecular markers of senescent cells include the following components of the SASP secretome: TNF, IL1b, IL6, IL8, IL11, CCL5, CXCL9, HSP60. In a young organism, SnCs exist for a limited time and play a crucial role in controlling oncogenic viruses. Additionally, markers of senescence include surface proteins such as B2MG, CD26 (DPP4) on mesenchymal cells, CD264 (TNFRSF10D, TRAILR4)—a decoy receptor for TRAIL on mesenchymal stem cells, Cathepsin F on fibroblasts and keratinocytes, uPAR on liver cells and macrophages, and DEP1 (PTPRJ/CD148) [52,58,59]. Markers predominantly expressed on SnCs in tumors have also been identified: DEP1, NTAL, EBP50, STX4, VAMP3, ARMX3, B2MG, LANCL1, VPS26A, and PLD3. For sorting SnCs from tumors, surface markers such as DEP1 and B2MG have been employed [60]. The identification of molecular markers of cellular senescence allows for the development of monitoring systems for aging and inflammation processes and their use in assessing treatment efficacy and testing substances with senolytic (SL—inducing selective elimination of SnCs) or senomorphic (SM) properties (blocking the negative effects of SnCs, such as cellular inflammation) [61]. The increase in SnCs numbers is also associated with reduced expression of endogenous reverse transcriptase inhibitors, which elevates systemic retrotransposon and retroviral activity, increasing the risk of DNA damage and triggering the activation of the senescence program. Cell cycle arrest in senescent cells occurs with elevated expression of p16 and p21, ultimately leading to the release of RB protein from the E2F complex [62]. During the unfolding of the senescence program, cells undergo epigenetic changes that are often characteristic of senescent cells up to their death [63]. The appearance of a large quantity of phosphorylated p53 leads to the activation of the p21 factor, promoting cell cycle arrest in the G0 phase; cells become “positive” for p16ink4 markers, and due to DNA damage, 53BP1 is recruited to the nucleus and initiates DDR [2,3]. In vivo, it was shown that the removal of p21/p16Ink4a-positive senescent cells in transgenic mice increased the animals’ average lifespan [64]. These two markers are the most studied, universal and reliable. The other described markers are auxiliary and illustrate the primary processes occurring in SnCs.: DNA damage, chromatin abnormalities, decrease in proliferation markers, erosion of nuclear envelope, activation of alarmins, prosecretory phenotypes, metabolic changes, oxidative damage and lysosomal changes [63].

## 4. Senescence and DNA Stability in Liver Pathology

The probability of DNA damage accumulation is higher in organelles, cells, tissues, and organs most exposed to negative effects such as, reactive oxygen species in mitochondria, genotoxins and catabolic products in kidney and liver [65]. Polyploidy and DNA copy number increase are intuitively considered to be a universal and widespread strategy in different organisms to compensate for negative mutagenic effects on cells. This universal strategy is observed in mitochondria, chloroplasts of eukaryotic cells, as well as in hepatocytes, kidney cells and, for example, trichomes in plants. Polyploidy can affect the level of transcription, but this connection is not general but is aimed at increasing the adaptive properties of the organism through activation induces pathways associated with stress response (hypoxia, oxidative stress, genotoxicity) and drug resistance, growth, DNA synthesis and ribosome biogenesis, while suppression of apoptosis genes, immune response, and aerobic metabolism is observed [66].

Polyploidisation and accumulation of genomic DNA damage have now been shown to mutually induce each other, and polyploid cells contain more genomic DNA damage than diploid cells, forming a reservoir of genomic damage, mitigating the effects of DNA damage [67] while enhancing its accumulation through delayed cell cycle arrest and reduced secretion of inflammatory cytokines associated with DNA damage-induced senescence [45,46].

The accumulation of polyploid cells is considered an unfavorable prognostic sign in the development of tumors, largely reflecting the organism’s compensatory mechanisms [68]. DNA copy number increase and carcinogenesis can be seen as a consequence of impaired maintenance of cellular genome integrity and stability [69,70].

In order to halt tumor progression senescence-inducing therapy is actively employed [9,71]. For instance, CDK4/6 inhibitors induce senescence and reduce tumor growth in breast cancer patients [72]. Cellular aging, an irreversible cell cycle arrest induced by the activation of DNA damage response pathways, acts as a crucial barrier to cancer development by suppressing the proliferation of abnormal cells [10,71].

Senescent cells play a key role in the pathogenesis of various metabolic diseases, including inherited metabolic liver diseases [65,73]. Hepatocytes are the type of cells in which compensatory mechanisms for genome preservation can most clearly and obviously operate. At the same time, hepatocytes serve as the primary target for gene therapeutic correction in various monogenic metabolic disorders, including hereditary hypercholesterolemia (*LDLR*), ornithine transcarbamylase deficiency (*OTC*), Crigler–Najjar syndrome (*UGT1A1*), hemophilia A (*F8*), hemophilia B (*F9*), glycogen storage disease type I (*G6PC*), and mucopolysaccharidosis types I, II, IIIA, and VI (corresponding to *IDUA*, *IDS*, *SGSH*, or *ARSB*), among others [71]. Hepatocytes are actively involved in metabolism and detoxification of the organism and also have a high ability to regenerate due to the regulation of proliferation and polyploidy, which makes them a convenient model for screening both senotherapeutic compounds and mechanisms of mutation fixation in mammalian somatic cells. With the development of CRISPR/Cas editing technologies, the development of any genotherapeutic product requires optimization of the fixation of introduced mutations or reversions into target cells, whereby, on the one hand, researchers have the opportunity to improve the editing system and, on the other hand, to expand the list of potential senolytic and senomorphic agents that can subsequently complement and improve treatment protocols for many cancer diseases.

Hemophilia B is one rather striking example illustrating that the nature of long-known, well-studied diseases may not be as obvious and well understood as previously thought. Hemophilia is extensively studied genetic disorder, first described in 1803 [72]. In 2022, the FDA approved the first gene therapy for hemophilia B, known as Hemgenix, which is based on an adeno-associated vector carrying the gene for coagulation factor IX. This gene is expressed in the liver to increase the levels of factor IX in the blood and reduce the risk of uncontrolled bleeding in hemophilia patients [74]. Current gene therapy delivery approaches utilizing adeno-associated virus (AAV) vectors face significant limitations: in actively dividing cells, the episomal vector is gradually lost, along with its therapeutic effect [75]. Consequently, these approaches are more suitable for adult patients whose hepatocytes are no longer characterized by frequent division. However, approximately 5% of adult patients with hemophilia B develop inhibitory antibodies against recombinant forms of FIX [76], highlighting the need for the development of universal gene therapy strategies using CRISPR-Cas editing of *F9*, employing non-viral delivery systems [77]. This would enable the reversion of the gene to a functional sequence at an early stage of disease development and the establishment of the patients’ immune systems. Despite the long history of studying and developing approaches for the therapy of hemophilia, there is still no universal approach to managing this disease and the medicines used are not without disadvantages and limitations. Thus, the data obtained in the study by Carpintero-Fernández et al. are intriguing; it was demonstrated that reducing levels of coagulation factor IX (FIX) prevents cell cycle arrest and the senescence-associated secretory phenotype (SASP) induced in tumor cells. While *F9* knockout prevents the induction of senescence, treatment with recombinant FIX halted the cell cycle and a senescence-like state in MCF7 tumor cells. Furthermore, various primary human cell cultures undergoing senescence exhibit elevated levels of endogenous FIX expression [78,79,80,81,82]. The absence of so-called “hot spot” regions in the *F9* gene, mutations (increased mutation frequency in exon 8 can be attributed to its larger size compared to other exons), the existence of sporadic mutations in the *F9* gene (30%), the presence of compound heterozygotes for *F9* mutations, as well as documented cases of secondary mutations in the *F9* gene upon deeper sequencing, suggest the possibility of an adaptive compensatory mechanism for the emergence of mutations at the *F9* locus [77]. Recently, it was shown that telomere length and mtDNA copy number in hemophilia patients are significantly lower than in healthy individuals. In patients with severe hemophilia, telomere length was greater than in those with mild hemophilia, while no differences in mtDNA copy number were observed [79]. Thus, hemophilia B may represent a unique model for studying the relationship between cell cycle regulation, genomic stability, and mammalian cell senescence.

One mechanism that buffers the accumulation of mutations in tissues where cells are most susceptible to factors negatively affecting genomic stability is polyploidy [80]. The processes of polyploidization and the induction of cellular senescence are closely related, as they are often observed concurrently [80,83]. However, the induction of cellular senescence is not necessarily accompanied by polyploidization; that is, polyploidization is not a prerequisite for cell cycle arrest: normal polyploid hepatocytes can proliferate vigorously to regenerate the liver [83]. It is known that adult human liver cells contain up to 40% polyploid hepatocytes, the number of which increases starting from the introduction of the first complementary foods to an infant and is regulated by dietary intake and living conditions. This indicates the presence of compensatory mechanisms within the liver that are activated in response to deteriorating environmental conditions and the complexity and decline of the dietary products consumed by humans. These mechanisms enable the liver to mitigate the adverse effects of environmental stressors on the organism. Although polyploid cells are genetically unstable and continue to accumulate mutations, these mutations are less likely to occur in a biallelic state compared to diploid cells. However, polyploid cells that retain the ability to proliferate may serve as a source of carcinogenesis as reservoirs of genomic anomalies. Indeed, it is believed that genome duplication during carcinogenesis occurs following genetic alterations in key cancer-associated genes [56,57,79,83,84,85]. Increased tolerance to the accumulation of genomic damage due to polyploidy may lead to the acquisition of resistance to chemotherapeutic agents, making the study of the mechanisms of fixation and homozygosity of mutations arising in polyploid cells particularly significant. It is particularly valuable that the acquired molecular mechanisms can be utilized to stabilize a population of hepatocytes with a wild-type sequence through contemporary genome editing approaches aimed at reverting target gene mutations.

One of the most critical criteria that cells assess at the cell cycle checkpoints is the presence of DNA damage [9,83]. In mice, it has been shown that livers deficient in Cdk1 exhibit significantly enlarged hepatocytes, accompanied by increased polyploidy [9,52]. The formation of “pathological” polyploidy in *Cdk1* knockout hepatocytes was halted upon the introduction of additional *CDK2* mutations, leading to the restoration of inflammation and reduced fibrosis in livers with double knockout of *Cdk1* and *Cdk2* [9,80]. In studies conducted on mouse liver and intestinal cells, it has been demonstrated that cellular senescence induced by a high-fat diet promotes the survival of cells harboring oncogenic mutations. Conversely, crizotinib, an inhibitor of HGF-mediated signaling, facilitates the elimination of these mutated cells. Thus, cellular senescence inhibits the competition-mediated elimination of oncogenic cells via HGF signaling, thereby increasing the likelihood of cancer development during the aging process [63].

The accumulation of senescent cells in brain tissue leads to the secretion of pro-inflammatory cytokines and other molecules that make up SASP, exacerbating neuroinflammatory processes and disrupting normal neuronal function [86]. These inflammatory changes may contribute to the progression of diseases such as Alzheimer’s and Parkinson’s, where chronic inflammation becomes a major contributor to neuronal death. Neurodegenerative diseases such as Alzheimer’s disease, Parkinson’s disease, amyotrophic lateral sclerosis, and Huntington’s disease are characterized by pathological reactivation of the cell cycle in post-mitotic neurons, which is associated with hyperactivation of Cdk5 kinase. Cyclin-dependent kinase Cdk5 is involved in signaling cascades responding to oxidative stress, DNA damage, and dysfunction of the ubiquitin-proteasome system. The activity of CDK5 is regulated by its activator p35, whose levels increase in aging and tumor cells. This interaction between CDK5 and p35 is essential for CDK5 activation, which, in turn, influences hepatocyte proliferation. Regulation of CDK5 depends on its activator p35, the expression of which increases in aging cells and tumor cells (osteosarcoma, prostate cancer, colorectal cancer) and is required for the activation of CDK5 induced by pRB during the unfolding of the senescence program. The p35-CDK5 complex phosphorylates MEK1, preventing phosphorylation of MAPK/ERK and cell proliferation [87]. Increased Cdk5 activity in aging cells leads to decreased activity of the Rac1 GTPase, which is necessary for actin polymerization, accompanying the morphological changes characteristic of cellular aging in response to pRb expression activated by Ras [88]. Inhibition of Cdk5 reduces the expression of the senescence marker SA-β-galactosidase and blocks actin polymerization. SA-β-gal is a biomarker used to identify senescent cells, which have permanently stopped dividing. It is an enzyme that accumulates in the lysosomes of senescent cells and can be detected at a specific pH (6.0) using a cytochemical or fluorescence-based assays [89]. Activation of Cdk5 is essential for inducing the senescent phenotype in cells expressing the pRB protein. The expression of pRB in tumor cells where it is absent leads to changes resembling senescence.

p35 negatively regulates NK cytotoxicity. The NK cytotoxicity inhibitor TGFβ induces the expression of p35. NK cells cultured with TGFβ demonstrate reduced cytotoxicity, while NK cells with *p35* knockdown or a mutant form of *CDK5* partially restore this inhibitory effect, indicating the important role of p35 and CDK5 in TGFβ-mediated NK exhaustion [90,91]. While it was previously thought that the influence of CDK5 was key only in the development and functioning of the nervous system, it has become evident over the past 20 years that CDK5 plays a significant role in regulating the differentiation of hematopoietic cells, immune responses, myogenesis, melanogenesis, insulin levels, cell migration, wound healing, invasion, cell survival, and angiogenesis. Dysfunction of CDK5 is associated with a range of diseases, including cancer, aging, diabetes, immune dysfunction, and inflammation. Combined approaches that integrate the modulation of signaling pathways involved in the regulation of the cell cycle and senescence, such as CDK5 and p35, with targeted therapy, CAR-T, immunotherapy, oncolytic viruses, and other innovative strategies may unveil new opportunities for the treatment of oncological and neurodegenerative diseases [60,92,93,94]. In the past decade, the search for drugs with SL activity, where specific cellular markers are used as therapeutic targets, has revealed the challenges in identifying a universal marker of senescence [95]. Senescent cells that accumulate in the liver with age activate signaling pathways such as the THBD–PAR1 axis, leading to impaired regeneration and fibrosis. Increased CDK5 activity in aging cells is associated with reduced Rac1 activity, which is necessary for actin filament polymerization, subsequently affecting the morphological changes characteristic of cellular aging [58]. CDK5RAP3 (CDK5 regulatory subunit-associated protein 3) plays a crucial role in liver development and function by controlling hepatocyte proliferation and differentiation, as well as glucose and lipid metabolism. Studies using *CDK5RAP3* knockout mice have demonstrated that the deficiency of this protein significantly impairs liver regeneration following partial hepatectomy, which is associated with delayed hepatocyte proliferation, increased lipid accumulation, and impaired glycogen synthesis. The knockout of *CDK5RAP3* reduces the expression of key metabolic genes, such as carnitine palmitoyltransferase 1α (Cpt1α) and fatty acid synthase (Fasn) [96]. Given that glucose metabolism and lipid oxidation provide essential energy sources after partial hepatectomy, these findings suggest a potential link between liver regeneration and glucose/lipid metabolism. Cyclin D1 restrains glucose uptake and glycogen synthesis through HNF4α-regulated metabolic adaptation, while the deletion of *HNF4α* in the liver induces Cyclin D1 expression and hepatocyte proliferation [97]. Today the findings highlight the complex interplay between lnc ATG9B-4, ARNTL, and CDK5 in liver cancer. The overexpression of *ATG9B-4* in liver cancer cells, leading to a downregulation of *ARNTL* and an upregulation of *CDK5*. *ARNTL* expression was negatively correlated with ATG9B-4 in liver cancer tissues, indicating that ATG9B-4 may inhibit *ARNTL* expression. The upregulation of ATG9B-4 leads to reduced ARNTL levels, which in turn results in increased *CDK5* expression, promoting the malignant characteristics of liver cancer cells, suggesting that targeting the ATG9B-4/ARNTL/CDK5 signaling pathway may provide new therapeutic strategies for managing liver cancer. Future research should focus on elucidating the direct regulatory mechanisms involved and exploring potential therapeutic interventions targeting this pathway to improve outcomes for liver cancer patients. The study underscores the significance of understanding LncRNA functions in cancer biology and their potential as therapeutic targets. ARNTL could downregulate *CDK5* expression in liver cancer cells. Knockdown of *CDK5* partially mitigated the proliferation and migration induced by ATG9B-4, suggesting that the ARNTL–CDK5 axis is crucial in mediating these effects. Transfection with *ATG9B-4* enhanced the proliferation and migration of HepG2 cells, while overexpression of *ARNTL* countered these effects. Specifically, the proliferation and migration rates were significantly lower in cells co-transfected with *ARNTL* compared to those transfected solely with *ATG9B-4*. Patients with low *ARNTL* expression exhibited poorer overall survival, reinforcing the idea that ARNTL plays a protective role in liver cancer progression [98].

These data underscore the intricate relationship between the regulation of regeneration and metabolic alterations following hepatectomy [99]. The coordination between cell cycle regulation and metabolic adaptation redirects intracellular resources to modulate proliferation during liver regeneration. The liver is the organ primarily responsible for detoxification of harmful substances. When studying the dynamics of cell cycle regulation (including the regulation of DNA stability maintenance and cell senescence) in response to negative physical or chemical factors, parallels can be drawn with the general biological strategies evolutionarily established by plants at the organismal level and by mitochondria at the cellular level. The established and evolutionarily conserved mechanisms include the enhancement of DNA copy number in response to increased environmental stress [83,100,101].

Cellular senescence and related mechanisms play a key role in the pathogenesis of many diseases, including cancer and neurodegenerative disorders. Understanding the molecular markers and signaling pathways associated with senescent cells opens new horizons for the development of therapeutic strategies. Senotherapeutic agents aimed at selectively eliminating (senolytics) or modulating (senomorphic) the activity and phenotype of senescent cells.

The search for factors and substances that can slow the progression of the chronic senescent phenotype is an exceptionally important task for extending working age, promoting active longevity, and preventing the early onset of age-related diseases.

## 5. Senotherapeutic Compounds

Due to sessile lifestyle, plants have evolved to produce a wide variety of primary and secondary metabolites (ScMs) as a crucial strategy for survival and stress protection. Despite the extensive use of various plant extracts in traditional medicine, the bioactive compounds within these extracts are often inadequately characterized. Approximately 50,000 ScMs have been documented in the literature, with the vast majority remaining untested for senolytic activity (SA) [102]. The ScMs that have been most thoroughly investigated for their ST are summarized in Table 1.

In addition to the compounds listed in Table 1, various substances and plant extracts with proven senolytic activity have been described, which have long been used by humans. Interest in studying their mechanisms of action has recently surged. For instance, one derivative of ellagitannin and ellagic acid, urolithin, has demonstrated significant senomorphic and senolytic activity in animal models [145,146,147]. The involvement of ellagitannin, ellagic acid, and urolithin in the stimulation of mitophagy and the restoration of normal energy metabolism in both human and animal cells has been demonstrated [148]. A primary limitation of senolytic therapy is the toxicity of senolytics towards non-senescent cells [149]. Urolithin consumption has been shown to be safe, even with a six-fold increase in plasma concentration [147]. The toxicity of potential senotherapeutic compounds is assessed using both bioinformatics approaches to select the most promising candidates and various in vitro and in vivo screening approaches [137,147,150]. Among these, the saponin oleandrin has been identified as a particularly promising senolytic, exhibiting biological activity at relatively low concentrations (10 nM), thereby reducing the risk of toxic effects during therapy with this compound [137]. Screening studies analyzing gene expression changes have identified senolytic substances (piperlongumine, phloretin, curcumin, and parthenolide) capable of functionally replacing the anticancer agent dasatinib, which is used in the treatment of myeloid leukemia. Notably, piperlongumine is considered an extremely promising senolytic due to its low toxicity [114].

Among plant-derived metabolites with senotherapeutic activity, there exists significant potential for the development of novel pharmaceuticals. However, despite promising results in preclinical models, several challenges persist in their translation to clinical practice. One of the primary issues is bioavailability, which can vary considerably depending on the chemical structure of the metabolite and its absorption capacity within the body. Metabolic stability is also a critically important factor, as many plant compounds may undergo rapid metabolism, thereby diminishing their therapeutic efficacy. Thus, while senolytics and senomorphic agents represent promising avenues in the fight against senescence-related diseases, their toxicity, specificity, and the challenges associated with translating plant-derived metabolites into therapies remain substantial hurdles that necessitate further research and development.

## 6. Biochemical Pathways Involved in the Accumulation of Senolytic Compounds: Potential Targets for Metabolic Engineering

The concept of metabolic engineering in plants has been around for approximately 50 years; however, with the advancement of CRISPR/Cas plant editing methodologies, this broad area of research is gaining renewed momentum and opens unique prospects for creating non-transgenic plants with enhanced levels of senolytic compounds to improve public health and quality of life [151,152,153].

### 6.1. Flavonoids Biosynthesis Pathway

**Figure 3:**
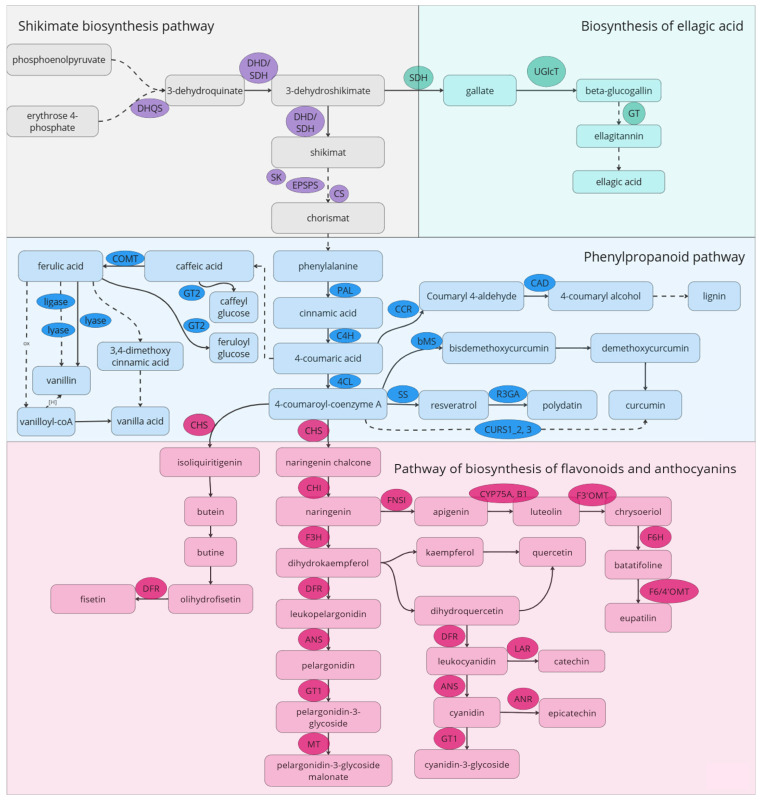
Key steps in the biosynthetic pathways of phenolic secondary metabolites in plants: Standard notations and abbreviations for the enzymes catalyzing the biosynthetic steps have been utilized (according to the KEGG database: https://www.kegg.jp/entry/map01110 (accessed on 24 June 2025)): DHQS—dehydroquinate synthase, DHD/SDH—dehydratase/shikimate dehydrogenase, SK—shikimate kinase, EPSPS—5-enolpyruvylshikimate-3-phosphate (EPSP) synthase, CS—chorismate synthase, UGlcT—UDP-glucose: gallate glucosyltransferase, GT—glycosyltransferase, COMT—caffeic acid/5-hydroxyferulic acid 3/5-O-methyltransferase, GT2—cinnamate beta-D-glucosyltransferase, PAL—phenylalanine ammonia-lyase, C4H—trans-cinnamate 4-monooxygenase, 4CL—4-coumarate-CoA ligase, CCR—cinnamoyl-CoA reductase, CAD—cinnamyl alcohol dehydrogenase, bMS—bisdemethoxycurcumin synthase, SS—stilbene synthase, R3GA—resveratrol 3-O-glycosyltransferase, CURS1,2,3—curcumin synthases, CHI—Chalcone isomerase, F3H—flavanone 3-hydroxylase, DFR—dihydroflavonol reductase, ANS—anthocyanidin synthase, GT1—glycosyltransferase 1, MT—malonyl CoA: flavonoid acyltransferase, LAR—leucoanthocyanidin reductase, ANR—anthocyanidin reductase, CYP75A, B1 flavonoid 3′,5′-hydroxylases, F3′OMT—flavonoid 3′-O-methyltransferase, F6H—flavonoid-6-hydroxylase, F6/4′ OMT—Flavonoid- 6/4′-O-methyltransferase. Dotted arrows indicate indirect synthesis, arrows indicate direct synthesis. Biosynthetic steps and enzymes that have already been validated in metabolic engineering research to redistribute metabolic pathways and accumulate more metabolite of interest are presented.

Polyphenolic compounds represent one of the largest and most widespread groups of plant metabolites with antioxidant, anti-inflammatory, senolytic, and senomorphic properties. Approximately 8000 phenolic compounds have been identified in plants, half of which are flavonoids, constituting two-thirds of the phenols consumed by humans [153]. The biosynthetic pathway of flavonoids is one of the most studied metabolic systems in plants (Figure 3). Previous attempts to modify flavonoid biosynthesis have been made to produce new pigmented flowers in ornamental plants and to enhance resistance against pathogens [154].

By regulating the expression levels and activity of transcription factors (TFs) and components of the rate-limiting steps in flavonoid biosynthesis, it is possible to significantly alter the levels of plant metabolites. Important regulatory families of plant TFs include MYB and MYC, which are part of the MBW regulatory complex, along with WRKY and NAC. Changes in the expression of these factors influence the accumulation of flavonoids and anthocyanins, assisting plants in adapting to biotic and abiotic stresses. In hops, constitutive expression of the TF HlMYB7 has been shown to repress flavonoid biosynthesis genes, while increased expression of the repressor HlMYB7 R2R3-MYB, conversely, leads to enhanced accumulation of these compounds [155]. Similar results have been observed in apple, rice, tomato, grape, persimmon, tea, strawberry, tobacco, and other plants, where not only the anthocyanin content in modified plants increased, but also the levels of flavonols and flavonoids [154].

The upregulation of key genes involved in flavonoid biosynthesis, such as *CHI*, *CHS*, *DFR*, *F3′5′H*, and *F3′H*, leads to an increased overall synthesis of secondary metabolites within this class in various plants, including rice, tomatoes, tobacco, legumes, and poplar [154,156,157,158,159,160,161,162,163]. Enhanced biosynthesis of flavonoids and flavonols, such as apigenin and luteolin, also promotes more effective recruitment of nitrogen-fixing bacteria, thereby improving plant yield and stress resilience [164]. Enzymes involved in anthocyanin biosynthesis, such as anthocyanidin reductase (ANR), anthocyanidin synthase (ANS), and leucoanthocyanidin reductase (LAR), are also associated with increased synthesis of flavonoids, alkaloids, and terpenoids [165,166]. Furthermore, mutations in the gene encoding the ubiquitin-like protein SDE2 in *Arabidopsis thaliana* resulted in enhanced expression of both regulatory genes and flavonoid and anthocyanin biosynthetic genes [167].

The most studied flavonoid-derived compound with senolytic activity is quercetin; quercetin and its glycosylated forms account for approximately 60–75% of the flavonoids consumed in the diet [168]. In *Arabidopsis*, it has been shown that mutant lines of the *HOS1* gene (encoding a ubiquitin ligase) negatively regulate the protein ICE1, which is targeted for degradation under cold stress, leading to increased expression of CBF-controlled *COR* genes. These lines were characterized by elevated levels of quercetin and kaempferol, conferring cold stress resistance to the plants [168]. Inactivation of the *OMT-1* gene, which encodes the enzyme that converts quercetin into isorhamnetin, results in the accumulation of quercetin and kaempferol [169,170]. Silencing of the *DFR* gene, which encodes dihydroflavonol-4-reductase, catalyzing the conversion of dihydroflavonol to anthocyanidin, led to a significant increase in quercetin levels due to the blockage of anthocyanidin biosynthesis [146].

### 6.2. Phenylpropanoid and Shikimate Pathways

Phenolic acids (PAs) represent a diverse group of metabolites. Examples of PAs include protocatechuic acid, p-coumaric acid, ferulic acid, sinapic acid, vanillic acid, hydroxycinnamic acid, caffeic acid, gallic acid, and ellagic acid [171].

In polymeric form, phenolic compounds exist in plants as lignins and tannins. Tannins in higher plants are classified into three classes: proanthocyanidins (condensed tannins), gallotannins, and ellagitannins (hydrolyzable tannins). Hydrolyzable tannins are less common in food products and beverages than condensed tannins [172]. There are two main classes of hydrolyzable tannins: gallotannins and ellagitannins. Acid hydrolysis of gallotannins yields sugar and gallic acid, while hydrolysis of ellagitannins produces sugar, gallic acid, and ellagic acid [108]. Gallotannins are the simplest hydrolysable tannins and are relatively rare in nature, found in mango and almond, in contrast to the more common ellagitannins found in fruits, nuts, and seeds [173]. Gallic acid is a key molecule for the biosynthesis of hydrolyzable tannins [174]. The accumulation of tannins or polyphenols can be modified by regulating the expression of genes encoding key enzymes in the biosynthesis of shikimic acid and ellagic acid. Gallic acid forms an ester with UDP-glucose [175], producing 1-O-galloyl-β-D-glucopyranose (β-glucogallin), which is the simplest gallotannin. Two UDP-glycosyltransferases (UGTs: PgUGT84A23 and PgUGT84A24) involved in the synthesis of β-glucogallin have been identified in pomegranate [151]. Knockout of UGT-encoding genes leads to an increase in the levels of gallic acids [176]. Under osmotic stress, two transcripts of shikimate dehydrogenase, PgSDH3s and PgSDH4, accumulated in pomegranate, resulting in an increase in the concentration of hydrolyzable tannins [177]. Similar results have been observed in tea, persimmon, grape, and olive [177,178,179,180]. One potential target for altering the accumulation of hydrolyzable tannins is the genes encoding tannase/tannin acyl hydrolases, which can break the ester bonds present in gallotannins and galloylated flavanols [181]. In strawberries, transient overexpression of tannase (FaTA) mRNA led to changes in ellagic acid levels; homologous sequences have been described in plants such as *Vitis vinifera*, *Juglans regia*, *Citrus clementine*, *Diospyros kaki*, and *Citrus sinensis* [182]. Regulatory cis-elements such as E-box and ARR1AT, which participate in the regulation of gene expression in response to brassinosteroids and cytokinins, respectively, have been found in the promoters of tannase genes in members of the *Juglandaceae* family. W-box and WUN motifs are involved in regulating gene expression in response to abiotic stressors such as damage. Thus, the expression of tannase genes in *Juglandaceae* may be controlled by signals related to phytohormones and responses to various environmental stress factors [182]. The regulatory regions of tannase genes in Chinese hickory (*Carya cathayensis*) and pecan (*Carya illinoinensis*) contain recognition motifs for transcription factors MYB and MYC [183]. Analysis of differential gene expression among different cultivars of Chinese olive, along with metabolic profiling, has identified key regulators of polyphenolic compound synthesis in these plants: chorismate mutase (ChM) and 3-deoxy-D-arabino-heptulosonate-7-phosphate synthase (DAHPS-1) [184]. In *Salvia miltiorrhiza*, editing the gene for rosmarinic acid synthase (SmRAS) resulted in a decrease in phenolic metabolites such as rosmarinic and lithospermic acids [162]. Knockout of the regulatory gene *bZIP2* led to an increase in the biosynthesis of phenolic acids [163]. Laccase enzymes are involved in the biosynthesis of phenolic acids; CRISPR/Cas-mediated knockouts of several genes in the *SmLAC* family in sage significantly reduced the biosynthesis of phenolic acids and lignin [185]. Similar results have been obtained in persimmon [186]. Silencing of the *AaC4H* gene *in Artemisia*, which encodes cinnamate-4-hydroxylase that converts trans-cinnamic acid to coumaric acid, resulted in increased trans-cinnamic acid levels and decreased levels of p-coumaric acid, salicylic acid, and the alkaloid artemisinin [187].

### 6.3. Alkaloid Biosynthesis Pathways

**Figure 4:**
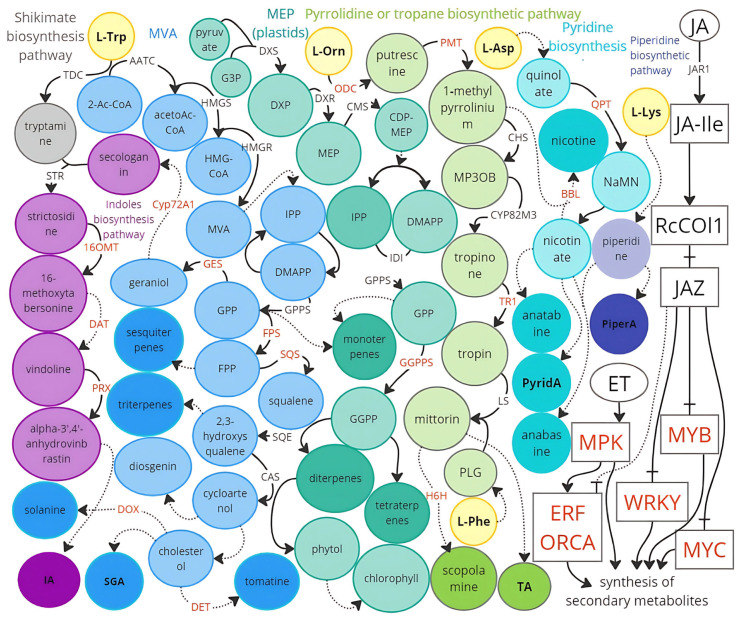
Major steps in the biosynthesis of alkaloids and pseudoalkaloids in plants: The diagram employs commonly accepted abbreviations for the names of enzymes catalyzing biosynthetic reactions and for intermediate compounds (according to the KEGG database, https://www.kegg.jp/entry/map01110 (accessed on 24 June 2025)). Enzyme names and transcription factors whose sequences have been already overexpressed or knocked out, resulting in altered profiles of plant secondary metabolites, are highlighted in red. A simplified schematic of the jasmonate and ethylene (ET) signaling pathways, which play a central role in regulating the biosynthesis of secondary metabolites of various types in plants, is also provided. Alkaloids are denoted as follows: IA—indoles, SGA—steroidal glycoalkaloids, TA—tropanes, PyridA—pyridines, PiperA—piperidines. An alphabetically organized list of abbreviations: 16OMT—16-O-methyltransferase; 2-AC-COA—Acetyl-CoenzymeA; AATC—acetyl-CoA acetyltransferase 1; acetoAcCoA—aceto acetyl-coenzymeA; BBL—Berberine Bridge Like genes; CAS—Cycloartenol synthase; CDP-MEP—4-Diphosphocytidyl-2-C-methyl-D-erythritol 2-phosphate; CHS—Chalcone Synthase; CYP72A1—cytochrome P450 dependent enzyme/secologanin synthase; CYP82M3—cytochrome P450 enzyme tropinone synthase; DAT—Deacetylvindoline O-acetyltransferase; DET—product of *SlS5αR2*, is responsible for the C5α reduction in α-tomatine biosynthesis; DMAPP—dimethylallyl diphosphate; DOX—*St16DOX* gene, encoding steroid 16α-hydroxylase; DXP—1-Deoxy-D-xylulose 5-Phosphate; DXR—MEP synthase; DXS—DXP synthase; ERF—Transcription factor; ET—Ethylene; FPP—Farnesyl Pyrophosphate; G3P—glyceraldehyde 3-phosphate; GES—Geraniol Synthase; GGPPS—Geranylgeranyl pyrophosphate synthase; GPP—Geranyl Pyrophosphate; GPPS—Geranyl Pyrophosphate Synthase; H6H—Hyoscyamine 6β-Hydroxylase; HMG—3-hydroxy-3-methyl-glutaryl; HMG-CoA—3-hydroxy-3-methyl-glutaryl-coenzyme A; HMGR—3-hydroxy-3-methyl-glutaryl reductase; HMGS—3-Hydroxy-3-Methylglutaryl-CoA Synthase; IDI—isopentenyl diphosphate isomerase; IPP—Isopentenyl Pyrophosphate; JA—Jasmonic Acid; JA-Ile—Jasmonoyl-Isoleucine; JAR1—catalyzes the formation of a biologically active jasmonyl-isoleucine (JA-Ile) conjugate; JAZ—jasmonate co-receptors; L-Asp—L-Aspartate; L-Lys—L-Lysine; L-Orn—L-Ornithine; L-Phe—L-Phenylalanine; L-Trp—L-Tryptophan; MEP—methylerythritol phosphate; MP3OB—4-(1-methyl-2-pyrrolidinyl)-3-oxobutanoic acid; MPK—Mitogen-Activated Protein Kinase; MVA—Mevalonic Acid; MYB—MYB transcription factors; MYC—MYC transcription factors; NaMN—3-Carboxy-1-[5-O-(hydroxyphosphinato)-α-L-arabinofuranosyl]pyridinium; ODC—Ornithine Decarboxylase; ORCA—Octadecanoid-Responsive Catharanthus AP2/ERF; PLG—pyridine based PLG (Pro-Leu-Gly-NH(2)) peptidomimetic; PMT—Putrescine N-methyltransferases; PRX—anhydrovinblastine synthase or peroxidase; QPT—Quercetin-3-Galactosyltransferase; Ras—RAS protein superfamily; RcCOl1—CORONATINE INSENSITIVE 1 (COI1), the recognition component; SGA—Steroidal glycoalkaloids; SQE—Squalene Epoxidase; SQS—Squalene Synthase; STR—Strictosidine synthase; TDC—Tryptophan decarboxylase; TR1—Tropinone reductase; WRKY—WRKY Transcription Factors. Indoles biosynthesis pathway—depicted in purple; Mevalonate (MVA) pathway—depicted in blue; Methylerythritol phosphate (MEP) pathway—depicted in emerald; Pyrrolidine and tropane biosynthesis pathway—depicted in apple green; Pyridine biosynthesis pathway—depicted in turquoise; Piperidine parts—depicted in royal blue; Classes of substances and amino acids are written in bold; The dotted lines show reactions through intermediate compounds; solid lines show direct reactions; A simplified diagram of hormonal regulation of secondary metabolite biosynthesis is shown in uncolored figures. The names of enzymes that have already been changed to modify biosynthesis of metabolites are written in red.

Metabolic engineering of alkaloid-rich crops can be aimed at both increasing their content for use as medicinal raw materials and reducing the alkaloid content and toxicity of plants to humans and livestock. The class of alkaloids is chemically extremely diverse: true alkaloids are defined as substances containing heterocycles in their structure, which are synthesized through amino acid biosynthetic pathways (Figure 4).

Among compounds with a confirmed alkaloid structure, true alkaloids include evodiamine (an indole alkaloid) and piperlongumine (a piperidine amide). The regulation of alkaloid biosynthesis genes in tobacco, rice, tea, and other plants involves transcription factors from the MYB, MYC, WRKY, GRAS families, as well as ATAF1-2, CUC2, HD-Zip, and CsHB1. The jasmonate signaling pathway (JA) plays a crucial role, as its induction leads to a surge in the production of secondary metabolites in plants, including alkaloids [186,188] (Figure 4). Indole alkaloids are synthesized from L-tryptophan, with their structure based on a bicyclic compound consisting of a benzene and a pyrrole ring. Approximately 2000 indole alkaloids have been described, among which caffeine (a purine alkaloid) and nicotine (a pyridine alkaloid) are the most well-known [189]. Using CRISPR/Cas technology, caffeine-free tea and nicotine-free tobacco have been produced by editing the genes *CsHB1* and *BBl*, respectively [190,191].

One of the promising indole alkaloids with senolytic activity is evodiamine, which is present in substantial quantities in *Evodia*, reaching up to 0.15% of the dry weight of the plant [192]. However, its content can be increased for greater industrial yields of indole alkaloids, drawing on previous studies involving the overexpression of genes from *Nothapodytes nimmoniana* and *Catharanthus roseus* that encode enzymes critical for the limiting steps in indole alkaloid biosynthesis, such as CrPrx, CrDAT, CrGES, CrGGPPS, NnCYP72A1, along with regulatory proteins CrMPK3, ORCA2, 3, 4, CrMYC1, OpWRKY2,3, CrERF5, and CrCR1 (AP2/ERF) from *Catharanthus roseus* and *Ophiorrhiza pumila* [187,193,194,195,196,197,198,199,200,201].

Pyridine alkaloids, including piperlongumine, are synthesized from L-ornithine in the biosynthetic pathways for tropane, piperidine, and pyridine alkaloids. Notable examples of increased concentrations of pyridine alkaloids in plants include studies on tobacco, where overexpression of *LcL/ODC* and *NtERF91* led to elevated levels of anabasine and anatabine [202,203]. In *Atropa belladonna*, knockout of *AbH6H*, which encodes hyoscyamine 6β-hydroxylase, resulted in increased synthesis of the tropane alkaloid hyoscyamine [204]. Overexpression of *DiH6H* (*Datura innoxia*), *SlTRI* (*Scopolia lurida*), *AbODC* (*Atropa belladonna*), and the transcription factor AbSAUR1 (*Atropa belladonna*), along with silencing of the *QPT* gene in *Duboisia leichhardtii*, successfully increased the content of tropane alkaloids [205]. A biallelic knockout of the *HSS* gene in *Symphytum officinale* led to a reduction in homospermidine or the entire fraction of pyrrolizidine alkaloids [206].

### 6.4. Pseudoalkaloid Biosynthesis Pathways

Pseudoalkaloids are heterocyclic compounds whose precursors are not amino acids but rather acetic acid, pyruvic acid, nitrogenous bases, or terpenoids. An example of this class of metabolites includes toxic steroidal glycoalkaloids, which play an important role in plant defense against pathogens and pests. To reduce the content of steroidal glycoalkaloids in tomatoes, a knockout in the *SlS5αR2 (DET)* gene was achieved through CRISPR/Cas editing, leading to the accumulation of the non-toxic precursor of the alkaloid alpha-tomatine. Knockout of the *StSSR2 (St16DOX*) gene in potatoes resulted in a decrease in the levels of steroidal glycoalkaloids in the aerial parts of the plant [207,208].

Terpenoids, or isoprenoids, constitute a vast class of pseudoalkaloids, comprising over 40,000 compounds. All terpenoids are synthesized through the mevalonate biosynthetic pathway and mevalonate-independent reactions [209]. Diterpenoids (e.g., 20-deoxygermacrene and oridonin), steroid saponins (proscillaridin A, digoxin, oleandrin, astragaloside), and sesquiterpenoids (dehydrocostus lactone) are synthesized from geranyl pyrophosphate (Figure 4). In *Catharanthus roseus*, overexpression of *CrMYC1* increased the content of terpenoid alkaloids, while five transcription factors (ORCA2,3,4,5,6) regulated the biosynthesis of terpenoid indole alkaloids, with ORCA4 enhancing the expression of genes involved in indole and iridoid alkaloid biosynthesis (e.g., morroniside) [198,210,211]. Silencing the squalene synthase gene (*SQS*) in *Artemisia* resulted in an increase in the biosynthesis of the sesquiterpenoid artemisinin [212]

## 7. Senotherapeutic Compounds and DNA Stability Maintenance

### 7.1. Resveratrol

Resveratrol, the most extensively studied secondary metabolite of plants, was first described 86 years ago [213] and has garnered increasing attention for its potential to prevent and suppress cancer. The consumption of small amounts of resveratrol is often cited as one explanation for the “French paradox”, which refers to the relatively low rates of cardiovascular disease mortality among the French population despite their high intake of butter, a staple of French cuisine. Recent studies have demonstrated that resveratrol inhibits the viability of breast and liver cancer cells and metastasis by enhancing the activity of SA-β-gal and modulating age-related molecular markers such as p53, p21, and Lamin B.

Additionally, *DLC1* further inhibited the DYRK1A–EGFR axis, leading to DNA damage characterized by elevated levels of the double-strand break marker γH2AX and reduced regulation of DNA repair proteins p-BRCA1 and RAD51, ultimately resulting in the senescence of cancer cells [214]. Low doses of resveratrol have been shown to accelerate non-mutagenic repair of DNA damage in mouse embryonic stem cells exposed to ionizing radiation. In mouse embryonic fibroblasts, resveratrol facilitates the efficient repair of double-strand breaks, thereby mitigating replicative stress and preserving genomic integrity [214]. Furthermore, resveratrol has been observed to significantly reduce DNA damage from arsenic compounds in non-cancerous mammalian cells by enhancing repair activities, particularly when administered prior to exposure. In cancer biology, resveratrol exhibits dual functionality; it can induce DNA damage while simultaneously activating repair mechanisms. In prostate, colon, and breast cancer cells, resveratrol has been shown to increase DNA damage, leading to cell cycle arrest and apoptosis [127,214]. Notably, in non-small cell lung cancer, resveratrol enhances the cytotoxic effects of pemetrexed by destabilizing the ERCC1 protein, a key player in DNA repair pathways [215]. Moreover, resveratrol has been reported to sensitize breast cancer cells to cisplatin by downregulating critical components of the homologous recombination pathway, thereby increasing susceptibility to DNA damage [202].

### 7.2. Curcumin

Curcumin, the active component of turmeric, is another phenolic compound with profound effects on DNA repair and genomic stability. Curcumin has demonstrated the ability to prevent DNA damage in lymphocytes exposed to arsenic, enhancing the repair capacity of base excision repair and non-homologous end joining pathways [203]. In murine models, curcumin reduces cyclobutane and pyrimidine dimers following UVB exposure, thereby delaying skin carcinogenesis [216]. In cancer cells, curcumin inhibits both homologous recombination and non-homologous end joining pathways by interfering with the acetyltransferase activity of CBP on histones at double-strand breaks [215]. Curcumin’s influence on DNA repair extends to its ability to induce DNA damage in gastric and breast cancer cells, activating the p53 pathway and suppressing cyclin E expression, which halts the cell cycle [217]. Furthermore, curcumin has been shown to modulate the expression of genes involved in DNA repair, such as *BRCA1* and *DNMT1*, ultimately leading to apoptosis in various cancer cell lines [218,219]. Its capacity to sensitize cancer cells to chemotherapy by affecting DNA repair pathways highlights curcumin’s potential as a therapeutic agent in cancer treatment.

### 7.3. Quercetin

Quercetin, a flavonoid abundant in fruits and vegetables, has been identified as a significant contributor to genomic integrity [220]. It has been shown to enhance the activity of base excision repair proteins, such as OGG1 and XRCC1, thereby reducing oxidative DNA damage in colon cells [221]. In prostate cancer cells, quercetin reduces the expression of key DNA repair proteins, including ATM and PARP1, and enhances radiosensitivity by blocking ATM activation [222,223]. The ability of quercetin to modulate DNA repair mechanisms has been further demonstrated in colorectal and breast cancer cells, where it acts as a radiosensitizer by prolonging damage persistence and inducing apoptosis [224]. By inhibiting BRCA2 activity during DNA replication, quercetin prevents efficient repair of double-stranded breaks, underscoring its potential as a therapeutic agent in cancer treatment [225].

### 7.4. Other Senotherapeutic Compounds

In addition to resveratrol, curcumin, and quercetin, other phenolic compounds such as kaempferol, genistein, and thymoquinone exhibit promising effects on genomic integrity and cell cycle regulation. Kaempferol has been shown to inhibit the expression of DNA repair proteins in leukemia cells while increasing phosphorylated p53 levels, suggesting its role in promoting apoptosis [226]. Genistein, derived from soy, has been reported to protect normal cells from ionizing radiation while inhibiting DNA repair pathways in cancer cells, enhancing their sensitivity to treatment [227]. Thymoquinone, found in *Nigella sativa*, induces DNA damage and apoptosis in glioblastoma cells, highlighting its potential as an anticancer agent [228]. The ability of these compounds to influence DNA repair mechanisms and enhance the efficacy of cancer therapies presents a compelling case for their inclusion in future research and clinical applications.

## 8. DNA Stability in Liver

By the ninth week of gestation, the fetal liver reaches its maximum relative size, constituting approximately 10% of the future child’s weight. During early gestation, the liver serves as the primary organ of hematopoiesis. At seven weeks of gestation, the number of hematopoietic cells surpasses that of hepatocytes. Morphologically, immature hepatocytes are smaller and contain less glycogen. The hepatic blood flow and the internal clearance of metabolites, which ensure the organism’s detoxification capacity, develop postnatally. The primary functions of the liver, including carbohydrate, lipid, and amino acid metabolism, detoxification of xenobiotics, serum protein synthesis, and bile production and secretion, are carried out by hepatocytes, which comprise up to 70% of the cells in the adult organ [218]. Hepatocytes exhibit functional heterogeneity, with different subtypes distributed along the porto-central axis, forming structural lobules. Periportal hepatocytes are responsible for gluconeogenesis, urea synthesis, β-oxidation of fatty acids, amino acid catabolism, and cholesterol biosynthesis, while pericentral hepatocytes are involved in glycolysis, lipogenesis, and detoxification [229,230].

Hepatocytes are also characterized by ploidy heterogeneity; from birth, they are diploid with a single nucleus and exhibit a high level of proliferation. During postnatal development, the liver parenchyma accumulates various classes of polyploid cells, with the proportion of polyploid cells in the human liver changing throughout ontogeny and differing from that of other mammals. Polyploid cells are generally larger in size, indicating hypertrophy. The dynamics of ploidy changes in liver cells during development have primarily been studied in rodents, demonstrating that hepatocytes cease active division relatively quickly, showing similar division dynamics to adult cells by the sixth week of development. Polyploidy in hepatocytes begins in the second week of development with the formation of tetraploid binucleated cells. Further cell proliferation can occur via various mechanisms and is influenced by developmental conditions, a phenomenon referred to as physiological polyploidy [231].

In studies of liver regeneration following hepatectomy, it has been shown that not all cells are equally involved in proliferation, with hypertrophied, polyploid cells being predominantly responsible. Polyploidy in healthy mammalian tissues is less common than in plants, insects, fish, or amphibians, occurring primarily in liver cells (4n, 8n), cardiac myocytes (4n), megakaryocytes (16n to 128n), giant trophoblasts of the placenta (8n to 64n), and in muscle and alveolar cells of mammary glands and acinar cells of the pancreas (4n) [216,217]. Polyploid cells (both nuclear and cellular polyploidy) normally constitute 30–50% of the hepatocytes in the adult human liver. In hepatocytes, ploidy is regulated by the number of nuclei per cell (typically one or two) and the DNA content in each nucleus: 2n, 4n, 8n, etc. Mononuclear hepatocytes can be diploid or polyploid, depending on the DNA content in a single nucleus: diploid (one nucleus 2n), tetraploid (one nucleus 4n), and so forth. Binucleated hepatocytes are always polyploid but can be tetraploid (two nuclei 2n), octaploid (two nuclei 4n), or higher. Various cellular mechanisms can induce polyploidy, including endoreplication, mitotic slippage, and cell fusion, with cytokinetic mitosis (also known as cytokinesis failure) being the dominant mechanism in healthy hepatocytes [218,219,220].

Polyploidization can occur through cell fusion (homo- and heterotypic) resulting in the formation of a syncytium (without nuclear fusion). The molecular mechanisms of cell fusion have been studied in detail, for instance, in myoblasts. Intercellular adhesion is crucial for fusion, which occurs in several stages: destabilization of opposing lipid bilayers, where myoblasts utilize actin membrane protrusions for the transport of specialized proteins and the formation of fusion pores. Cell fusion also plays a key role in wound healing and tissue regeneration. Viral infections can also significantly contribute to the formation of polyploid cells. The most well-studied example of such influence is human papillomavirus (HPV): upon integration of the virus into the human genome, the expression of the oncogene *HPV-16 E5* leads to the fusion of epithelial cells, resulting in the formation of tetraploid binucleated cells, which serve as a negative prognostic indicator for precancerous conditions of the cervical epithelium [232,233,234,235].

Polyploid cells can also arise from endoreplication in the absence of cytokinesis, resulting in mononuclear polyploid cells. Endoreplication can be classified into endocycling and endomitosis. In endocycling, cells undergo alternating G and S phases of the cell cycle, while in endomitosis, cells are arrested at metaphase, anaphase, or cytokinesis. The defining event in the initiation of endocycling is the inhibition of mitosis initiation through the proteolysis of cyclins by the E3 ubiquitin ligase APC/C (anaphase-promoting complex; also known as the cyclosome), or the suppression of M-CDK by inhibitors. Another characteristic feature preceding endocycling is the oscillation of S-CDK activity from low to high during G and S phases: the accumulation of cyclin-dependent kinase inhibitors p57 and p21 is necessary for the induction of endocycling, as this leads to the degradation of cyclin B and suppression of CDK1 activity [236]. During endoreplication, genomic instability is often observed: for example, the induction of UV-induced double-strand breaks in *Arabidopsis thaliana* leads to G2–M cell cycle arrest, and prolonged damaging UV exposure initiates endoreplication [236]. In human and mouse cell lines characterized by a high number of defective telomeric chromosomal regions, p53/pRb-induced endoreplication is observed. Prolonged G2 arrest results in the formation of mononuclear tetraploid cells [237]. Endomitosis is often observed, when the transition from metaphase to anaphase is delayed; many antimitotic drugs used in cancer treatment target microtubule dynamics and block spindle formation. The regulation of the transition to anaphase and the initiation of spindle assembly is controlled by the proper attachment of all chromosomes to the kinetochore. Persistent activation of the spindle assembly checkpoint can lead to either cell death due to “mitotic catastrophe” (resulting in cancer remission) or the exit from mitosis in metaphase, also known as mitotic slippage, which contributes to cancer progression. This process is regulated by the antagonistic activity of apoptotic components and two mitotic ubiquitin ligases [238]. Mitotic “slippage” is associated with the proteolysis of cyclin B1 by the APC/C and CRL2ZYG11A/B ubiquitin ligases. It remains unclear why cells exposed to drugs that block spindle formation evade apoptosis; it is believed that this is largely due to the evolutionarily developed resistance of polyploid cells to various stresses and insults. Mutations in the *APC* (adenomatous polyposis coli) gene associated with colorectal adenocarcinoma have been described. APC is a component of the WNT signaling pathway and is associated with the microtubule cytoskeleton; the absence of APC leads to chromosomal instability due to improper attachment of chromosomes to the kinetochore, resulting in the formation of mononuclear tetraploid cells [239,240]

Cytokinesis failure, initiated upon completion of anaphase, also frequently leads to the formation of polyploid cells. The initiation of cytokinesis occurs after the cell undergoes sequential reorganizations: formation of the division axis (cell polarization), formation of the actomyosin ring, deepening of the furrow, and formation of an intracellular bridge, the degradation of which leads to cell separation. Cytokinesis failure is often observed during normal tissue formation in the bone marrow, heart, and liver. For instance, ventricular cardiomyocytes respond to increased blood flow after birth with adaptive volume hypertrophy. The transition from hyperplasia to hypertrophy is associated with polyploidy: the formation of binucleated tetraploid cardiomyocytes. The degree of polyploidy in ventricular cardiomyocytes varies with age and between species: in rodents, the percentage of polyploid cells can reach 85%, while in humans, this percentage is lower, reaching 30–50% [238,241]. In the regulation of polyploidy in cardiomyocytes, cyclin G1 plays a crucial regulatory role, with its expression observed during the transition of neonatal cells from G1 to S phases of the cell cycle, while cytokinesis is inhibited. When cytokinesis is blocked in cardiomyocytes, altered localization of contractile ring proteins, such as RhoA, ROCK-I, ROCK-II, and anillin, is also observed [241]. Cytokinesis failure is characteristic and frequently observed in the development of various diseases, often associated with high mutation rates and genetic instability. Even disruptions in chromatin compaction, leading to the formation of larger structures or delays in chromosome arm movement during cell division, can result in cytokinesis failure and the formation of tetraploid cells in human tissues [242]. Cytokinesis failure is also frequently observed in tumor cells during the development of breast cancer, colorectal cancer, and ovarian cancer, where mutations in *aurora A*, mitotic arrest-deficient 2 (*MAD2*), and *LATS1* (large tumor suppressor 1) have been documented [243].

**Figure 5:**
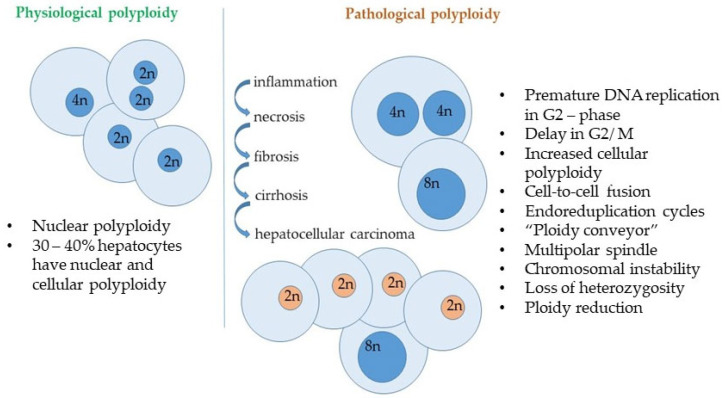
Physiological and pathological hepatocytes polyploidy differences.

The biological significance of polyploidy can vary; its role in maintaining genomic stability through regeneration and repair, as well as in regulating organ size and regeneration in the liver, kidneys, and heart, has been demonstrated [244,245,246]. Chromosome doubling is associated with increased hepatocyte size. The accumulation of polyploid cells is linked to many age-related diseases in humans, including hyperthyroidism, oncological diseases, and arterial hypertension [244,245,247]. The proliferation of polyploid cells often leads to genetic instability and is associated with oncological diseases (Figure 5). In the liver, a slightly different situation is observed: hepatocyte proliferation can be induced by substances such as laminin and fibronectin [248,249]. The primary factor stimulating hepatocyte proliferation is the growth factor HGF. Proteoglycans in the hepatic extracellular matrix play a key role in regulating the expression and binding availability of HGF, with some significantly enhancing the mitogenic activity of cells (heparin, chondroitin, heparan sulfate proteoglycans, perlecan, syndecan, decorin) [250,251], while others bind HGF with lower affinity (GPC3), thus regulating the activity of the growth factor [252,253]. The expression of heparan sulfate proteoglycans is activated after injury, increasing HGF activity and mitogenic activity in the liver. The quantity and ratio of proteoglycans direct the processes of liver regeneration and organogenesis: during hepatectomy, there is an increase in the levels of another growth factor, TGFβ, a negative regulator of hepatocyte proliferation and an activator of proteoglycan expression (such as decorin and syndecan) [254,255,256].

Studies in rats have shown that the number of polyploid hepatocytes increases with age, while at early stages of organogenesis, diploid and tetraploid hepatocytes are present in approximately equal proportions. Furthermore, mechanisms supporting wild-type alleles in tetraploid cells are hypothesized to exist, with markers of senescent hepatocytes (e.g., Cyp2e1) predominantly expressed in tetraploid cells, suggesting that hepatocyte polyploidy may serve as a buffering mechanism against age-related changes [257,258].

Diploid hepatocytes accelerate liver regeneration following hepatectomy and may enhance compensatory regeneration after acute injury. Polyploid hepatocytes protect the liver from tumorigenesis in hepatocellular carcinoma (HCC) and contribute to adaptation to chronic damage caused by tyrosinemia, for example [256].

Studies of liver regeneration after hepatectomy have shown that not all cells are equally involved in proliferation, with hypertrophied polyploid cells playing a dominant role. Polyploidy is less common in healthy mammalian tissues than in plants and insects, and is mainly observed in liver cells, cardiac myocytes, and megakaryocytes. Polyploid cells (both nuclear and cellular) typically comprise 30–50% of hepatocytes in the adult human liver.

Hepatic stellate cells, also known as Kupffer cells, are mesenchymal cells located in the space of Disse. They make up approximately 8% of all liver cells and store between 70% and 95% of all retinoid lipids (vitamin A) in the homeostatic liver. Hepatic stellate cells play a critical role in hepatocyte proliferation by secreting important signaling molecules such as HGF. Following partial hepatectomy, HGF stored in the extracellular matrix is activated and transforms into a heterodimeric form capable of binding to the MET receptor on hepatocytes via a paracrine pathway and through the peripheral circulation, activating several downstream signaling pathways that facilitate the G1/S transition of the cell cycle. Excessive fibroblast growth factor (FGF)-10 expression induced by hepatic stellate cells also affects hepatocytes, promoting their proliferation during ischemia–reperfusion injury (IRI). Hepatic stellate cells are primarily stimulated to produce HGF by interleukin-6 (IL-6) trans-signaling, highlighting the critical role of the soluble IL-6 receptor (sIL-6R) in liver recovery. In addition, hepatic stellate cells are involved in the termination of liver regeneration by contributing to the reconstitution of the extracellular matrix, which isolates hepatocytes from growth factors and induces proliferating hepatocytes to exit the cell cycle. TGF-β secreted by hepatic stellate cells inhibits the regenerative response of hepatocyte proliferation during the termination phase. Hepatic stellate cells also have the potential to replenish liver mass by differentiating into hepatocytes and bile duct cells under certain conditions, with the possible mechanism involving transdifferentiation via the Hippo/Yes-associated protein (YAP) pathway [257].

With increasing age, senescence becomes an important factor in liver regeneration. Aging disrupts the effective regenerative process through p53 and p21-mediated cell cycle arrest, which halts hepatocyte proliferation. Telomere shortening, another hallmark of aging, exacerbates the senescent phenotype, reflecting cumulative cellular damage over time and impairing liver function in chronic disease. The liver of the elderly exhibits reduced regenerative capacity, which is also associated with epigenetic changes, particularly those involving CCAAT/enhancer binding protein alpha (C/EBPα). These changes inhibit the expression of key regenerative genes, thereby compromising the liver’s ability to recover from injury. While senescent cells initially act as a tumor suppressive mechanism, they can contribute to a pro-inflammatory environment that hinders regeneration. The senescence-associated secretory phenotype (SASP) is characterized by the secretion of several pro-inflammatory cytokines and growth factors that can adversely affect neighboring non-senescent cells, promoting a cycle of chronic inflammation and fibrosis. This phenomenon highlights the dual nature of senescence in the liver, where it can both protect against tumorigenesis and impair regenerative processes. Furthermore, the interplay between senescence and liver fibrosis is increasingly recognized, as senescent hepatic stellate cells (HSCs) contribute to the fibrogenic response. Activation of HSCs leads to excessive extracellular matrix production that disrupts normal liver architecture and function. Interventions aimed at modulating senescence and its associated pathways may offer new therapeutic avenues to enhance liver regeneration, particularly in the aging population [258]. The intricate interplay between hepatocyte biology, genomic integrity, and cell cycle regulation positions hepatocytes as a convenient model for studying the effects of senolytic compounds. Chronic cellular senescence is a known mediator of tissue function decline in aging and disease. Understanding the complex relationship between aging, senescence, and liver regeneration is crucial for the development of effective therapeutic strategies. The identification of specific molecular targets and pathways involved in these processes may pave the way for novel treatments aimed at improving liver health and function in older adults.

In mice with steatohepatitis and fibrosis, disorders with metabolic dysfunction in the liver, a reduction in disease progression and the risk of developing hepatocellular carcinoma was observed due to proteolysis of BCL-xL/BCL-2 when using a liver-tropic senolytic 753b with increased sequestration in the organ [259].

Natural substances with serotherapeutic activity are less toxic compared to synthetic analogs [149]. As examples, the action of the most studied senolytics can be considered; thus, resveratrol has been shown to help prevent non-alcoholic fatty liver disease (NAFLD) by regulating lipid levels and activating the AMPK/SIRT1 axis. It reduces fat accumulation in the liver by increasing fatty acid oxidation and decreasing lipogenesis. Resveratrol has significant antioxidant properties, reducing levels of reactive oxygen species (ROS) and enhancing the activity of endogenous antioxidants such as superoxide dismutase (SOD) and catalase (CAT). It activates signaling pathways, including AMPK/SIRT1/Nrf2, which facilitates the synthesis of antioxidant molecules. Resveratrol reduces oxidative stress and inflammation, improving outcomes after liver transplantation. In liver fibrosis, resveratrol activates hepatic stellate cells, leading to a reduction in portal pressure and improved endothelial function in cirrhotic models. In experimental models of cholestatic liver injury, resveratrol reduces levels of pro-inflammatory cytokines such as TNF-α and IL-6, while promoting hepatocyte proliferation. In models of hepatocellular carcinoma and its metastasis, resveratrol induces apoptosis in tumor cells by activating both extrinsic and intrinsic pathways. It inhibits tumor-associated signaling pathways such as PI3K/Akt/mTOR and NF-κB. Resveratrol restores glucose regulation in the liver by decreasing the activity of key gluconeogenic enzymes and increasing glycogen synthase activity. Resveratrol also protects the liver from the toxic effects of substances such as cadmium and naphthalene by reducing oxidative stress and preventing cell damage. Overall, the multiple effects of resveratrol on liver health underscore its potential as a therapeutic agent in the treatment of liver disease, highlighting its role in reducing inflammation and oxidative stress and promoting cellular regeneration. Further research is warranted to fully elucidate its mechanisms of action and to optimize its therapeutic applications in clinical settings [126]. Another senolytic compound curcumin has been shown to reduce the number of senescent cells in the liver and other organs, leading to improved functional parameters and reduced inflammation. For example, in MAFLD models, a reduction in inflammatory markers and an improvement in the metabolic state of the liver were observed following curcumin administration. These findings highlight the potential of curcumin as a therapeutic agent in the treatment of liver diseases, particularly those associated with aging and metabolic dysfunction [260]. Quercetin, in turn, induces apoptosis in various cell types, including liver cells, by disrupting the balance between pro-apoptotic and anti-apoptotic proteins. It reduces the levels of the anti-apoptotic protein Mcl-1 while activating the pro-apoptotic protein Bax. This leads to the release of cytochrome c from the mitochondria and the activation of caspases, resulting in cell death. Quercetin also stimulates autophagy by activating signaling pathways such as AMPK and mTOR. It enhances lysosomal biosynthesis and facilitates the removal of damaged cellular components, which may help protect the liver from injury [256]. Additionally, quercetin inhibits RIPK1 activity, preventing necrosome formation and reducing inflammatory responses, making it a promising agent for combating necroptosis. It modulates pyroptosis by inhibiting the activation of the NLRP3 inflammasome and reducing levels of pro-inflammatory cytokines such as IL-1β. Quercetin allows cells to exhibit protective properties against ferroptosis by decreasing oxidative stress and regulating iron metabolism. It binds to iron and mitigates its toxicity, thereby helping to prevent cellular damage. Furthermore, quercetin may influence cuproptosis by binding to copper and reducing its toxicity, opening new avenues for treating diseases associated with copper dysregulation. While fibrosis resulting from chronic liver injury, quercetin inhibits the activation of hepatic stellate cells (HSCs) and prevents fibrosis progression. It reduces collagen production and diminishes inflammation, which may also slow the progression of fibrosis in the organ. Quercetin acts on key signaling pathways, such as TGF-β1/Smads and PI3K/Akt, which are associated with HSC activation and collagen synthesis. This positions quercetin as a promising therapeutic agent for liver diseases related to fibrosis [261].

Somatic mutations accumulate in various organs and tissues during the aging process. A comparison of the somatic mutation burden between early passage and deeply senescent human fibroblasts using single-cell whole-genome sequencing revealed that senescent cells exhibit 2.4 times more single nucleotide variants (SNVs) and 1.9 times more insertions and deletions (INDELs) compared to early passage cells. This increase in mutation frequency can be attributed to the greater number of cell divisions that senescent cells are likely to have undergone. The mutation spectra of SNVs and INDELs did not show significant differences between early passage and senescent cells, indicating the absence of specific mechanisms for generating mutations in senescent fibroblasts. In contrast to SNVs and INDELs, aneuploidies were detected exclusively in senescent cells. This suggests that aneuploidy may be associated with the process of cellular senescence [262]. While senescent cells exhibit an increased mutation load, the mechanisms underlying this accumulation warrant further investigation. These findings underscore the importance of analyzing mutational burden in the context of aging and diseases related to both cellular senescence and mitochondrial dysfunction. Senolytics remove senescent cells that accumulate mutations and contribute to inflammation and other pathologies. Removing these cells can lead to a reduced mutation load in tissues and improved cellular function, which in turn can slow the aging process and reduce the risk of developing age-related diseases.

## 9. Conclusions

Contemporary medicine and science face a challenge that necessitates a reevaluation of traditional approaches to diagnosing and treating diseases. Specifically, relying solely on symptomatic presentations or the localization of inflammation is no longer a sufficient basis for making diagnoses. A deeper understanding of the underlying mechanisms of diseases is essential for effective management. Specifically, the example of hemophilia B illustrates how the coagulation factor IX (F9) is implicated in cellular senescence, suggesting that mutations in the *F9* gene may reflect broader pathological processes. Recent research indicates that coagulation factor IX plays a critical role in the process of cellular senescence, suggesting that sporadic mutations in the *F9* gene may compensate for and reflect other pathological processes within the body. It has recently been shown that hemophilia patients show signs of accelerated biological aging, as evidenced by significantly shorter telomeres and lower mtDNA copy numbers compared to healthy controls [58]. The findings align with existing literature indicating that shorter telomeres are associated with various chronic diseases and may reflect increased biological aging. The study posits that the observed biological aging in hemophilia patients could result from chronic inflammation, oxidative stress, and the effects of hemophilia-related complications. These findings underscore the need for further investigations into the mechanisms of biological aging in hemophilia and the potential implications for treatment and management strategies. This example underscores the importance of investigating the molecular mechanisms underlying pathology, rather than solely their clinical manifestations. Another interesting example of revision of the established ideas about the molecular nature of liver function and diseases is the protein kinase *CDK5* and its still not fully understood contribution to the regulation of liver function. The possibility of using liver cells to screen for senolytic agents may not only provide a route to the development of improved approaches to the treatment and prevention of cancer recurrence, and in the long term may help to educate the general public about healthy eating habits but may also lead to the development of tools aimed at increasing the efficiency of CRISPR/Cas9 technologies by selecting optimal conditions for fixing the introduced mutations in the population of cells. This approach has the potential to significantly advance our understanding of mammalian DNA repair mechanisms and facilitate targeted interventions for a variety of genetic disorders, thereby contributing to the broader field of gene therapy and regenerative medicine. The intricate interplay between hepatocyte biology, genomic integrity, and cell cycle regulation positions hepatocytes as a convenient system for studying senolytic compounds. Hepatocytes not only serve as a critical model for elucidating the mechanisms underlying liver regeneration and disease but also offer a promising platform for investigating novel therapeutic strategies. The ongoing exploration of these pathways could yield significant insights and therapeutic advancements in managing liver-related diseases and enhancing regenerative capacities that could transform the treatment of chronic diseases.

## Figures and Tables

**Table 1:** Secondary metabolites with senolytic or senomorphic activity.

Compound	Plants	ST	Targets	References
Epigallocatechin gallate ^1^	Green tea	SM	↓ PI3k/Akt/mTOR, ROS, Cox2, NF-κB, IL-6, TNF-α and ↑ AMPK	[103]
Apigenin ^1^	*Lamiaceae*	SM	AMPK-mTOR-TFEB, NF-κB subunit p65 and IκB	[104]
Eupatilin ^1^	Wormwood	SM	MAPK-NF-κB, ↓ *e* p21, p53	[105]
Kaempferol ^1^	Apples, grapes, tomatoes, green tea, etc.	SM	MAPK, NF-κB subunit p65 and IκB	[106]
Luteolin ^1^	Celery, parsley, broccoli, apple peels, chrysanthemum flowers	SM	↑ SIRT6, ↓ NF-κB	[107]
Proanthocyanidins ^1^	Fruits, bark, leaves, seeds of many plants	SM	↓ PI3K-Akt	[108,109]
Anthocyanins ^1^	Canadian elderberries	SL	↓ PI3K/AKT/mTOR	[109]
Quercetin ^1^	*Rosaceae*, *Theaceae*, *Brassicaceae*, *Asparagaceae*, *Ericaceae*, *Moraceae*	SL SM	↓ NF-κB, ↑ SIRT-1, ↓ COX and lipoxygenase; mTOR, PI3K/Akt, p53/p21/serpins	[4,110]
Butein ^2^	Dahlias, coreopsis	SM	Sirt1-p53	[111,112]
p-Coumaric acid ^2^	Peanuts, beans, tomatoes, sweet clover, carrots, basil, garlic, strawberries	SM	↓ Nrf2-NF-κB	[113]
Gallic ^2^ and ellagic acids ^2^	Blackberries, cloudberries, pomegranates, raspberries, strawberries, chestnuts, walnuts	SL	↓ *e* BCL-2, ↓ NF-κB, ↓ TNFα, ↓ IL-1β, ↓ IL-6, ↓ ROS	[48]
Curcumin ^2^	Turmeric	SM	AMPK-mTOR-ULK1↑	[114]
		SM SL	p70/S6K, Akt-LC3-II-SQSTM1/p62 JNK, ↑ Nrf2, ↓ NF-κB, and *e* pro-inflammatory cytokines, ↓ *e* IL-1β, TNF-α, IL-10	[115]
Fisetin ^2^	Lacquer tree (*Anacardiaceae*), strawberries, apples, persimmons, grapes	SL SM	↑ SIRT1, ↓ IL-6, TNF-alpha, ↓ NF-κB and Nrf2	[116,117]
Honokiol ^2^ (“hou po”)	Bark and leaves of magnolias	SM	↑ AMPK-PGC-1α-SIRT3	[118]
Myricetin ^2^	*Myricaceae*, *Polygonaceae*, *Primulaceae*	SM	SERPINE1 SIRT1-PGC-1α, ↓ IL-1β and ↓ IL-6, ↓ *e* p21, p16	[119,120]
Polydatin ^2^	Vitaceae, Liliaceae, Fabaceae	SL	Nrf2-HO-1	[121]
Resveratrol ^2^	Grapes, raspberries, mulberries, pistachios, and peanuts	SM SL	SIRT1, ROS-NF-κB ROS-PI3K-Akt	[122,123,124,125,126,127]
Vanillin ^2^	Vanilla orchid, Korean pine fruits, mango	SL SM	TLR-2, NF-κB, Nrf2, ↓ SASP	[128,129]
Gingerol A, 6-Shogaol	Ginger	SL	Caspase-3, ↓ Bcl-XL, ↓ IL-6	[100]
Avenanthramide C ^2^	Oats, *Isatis tinctoria* L. leaf extract	SL SM	↓ p21 CDKN1A and p16INK4A, SASP, ↑ AMPK, ↓ mTOR, MAPK, and IκBα, ↓ NFκB	[130]
Dehydrocostus lactone ^4^	Costus, sunflower	SM	STING-TBK1-NF-κB, MAPK	[131]
Evodiamine ^3^	Dried immature fruits of *Evodia*	SM	Nrf2-HO-1, MAPK	[132]
Piperlongumine ^3^	Long pepper (*Piperaceae*)	SL	Inactivation of OXR-1 and ROS-reducing enzyme	[114,133]
20-Deoxyingenol ^4^	Bark of *Erythroxylum* tree	SM	TFEB-mediated autophagy	[134]
Kinsenoside ^4^	Anectochilus (*Orchidaceae*)	SM	Akt-ERK1/2-Nrf2	[135]
Morroniside ^4^	Cornelian cherry	SM	ROS-Hippo-Mst1/2 and Lats1/2-YAP/TAZ-p53	[108]
Proscillaridin A ^4^, Digoxin ^4^	Wooly and purple foxglove	SL	Na^+^/K^+^ATPase, apoptosis	[136]
Oleandrin ^4^	Oleander	SL	↑ *e* NOXA, ↓ p16 and p21, and pro-inflammatory cytokines IL1α, IL1β, and IL8	[137]
Astragaloside ^4^	*Astragalus membranaceus*	SL	STING/NF-κB	[138]
Oridonin ^4^	*Lamiaceae*	SL SM	↓ IL-6 and IL-8, ↓ p38, p65 (NF-κB), glutathione S-transferase	[139,140,141]
Active substance not identified	Common goldenrod	SL	↓ SASP	[142]
Cocktail of substances	Fruits of *Terminalia chebula* (haritaki)	SL SM	↓ CSF3, CXCL1, IL-1β, IL-6, and IL-8, ↓ miR 29a-3p, miR 30a-3p, miR 34a-5p, miR 24a-3p	[143]
Active substance not identified	Extract of flowers of *Silybum marianum*	SL	↓ IL-6, MMP-1	[144]

^1^ Flavones and flavonoids, anthocyanins; ^2^ Phenolic compounds from shikimic and phenylpropanoid biosynthetic pathways; ^3^ Alkaloids (indoles, piperidines, and pyridines); ^4^ Pseudoalkaloids: steroid saponins (biosynthetic pathway of mavolanates and methylerythritol phosphates), glycosides (glycopyranoside, iridoid glycoside), terpenes (biosynthetic pathway of terpenoids); “*e*”—expression, “↓”—decrease, “↑”—increase, ST—senotherapeutic activity, SL—senolytic activity, SM—senomorphic activity.

## Data Availability

Data are contained within the article.

## Abbreviations

The following abbreviations are used in this manuscript:

SnCs	Senescent cells
ScMs	Secondary metabolites
ST	Senotherapeutic activity (senolytic or senomorphic)
SL	Senolytic activity or compound with senolytic activity
SM	Senomorphic activity or compound with senomorphic activity
